# The role of ncRNAs and exosomes in the development and progression of endometrial cancer

**DOI:** 10.3389/fonc.2024.1418005

**Published:** 2024-08-12

**Authors:** Julia Niebora, Sławomir Woźniak, Dominika Domagała, Krzysztof Data, Maryam Farzaneh, Mojtaba Zehtabi, Mahrokh Abouali Gale Dari, Fatemeh Khojasteh Pour, Artur Bryja, Magdalena Kulus, Paul Mozdziak, Piotr Dzięgiel, Bartosz Kempisty

**Affiliations:** ^1^ Division of Anatomy, Department of Human Morphology and Embryology, Faculty of Medicine, Wroclaw Medical University, Wroclaw, Poland; ^2^ Fertility, Infertility and Perinatology Research Center, Ahvaz Jundishapur University of Medical Sciences, Ahvaz, Iran; ^3^ Clinical Research Development Unit, Imam Khomeini Hospital, Ahvaz Jundishapur University of Medical Sciences, Ahvaz, Iran; ^4^ Hematology and Oncology Research Center, Tabriz University of Medical Sciences, Tabriz, Iran; ^5^ Department of Obstetrics and Gynecology, School of Medicine, Ahvaz Jundishapur University of Medical Sciences, Ahvaz, Iran; ^6^ Department of Obstetrics and Gynecology, School of Medicine, Hamadan University of Medical Sciences, Hamadan, Iran; ^7^ Department of Veterinary Surgery, Institute of Veterinary Medicine, Nicolaus Copernicus University in Torun, Torun, Poland; ^8^ Physiology Graduate Program, North Carolina State University, Raleigh, NC, United States; ^9^ Division of Histology and Embryology, Department of Human Morphology and Embryology, Faculty of Medicine, Wroclaw Medical University, Wroclaw, Poland; ^10^ Department of Obstetrics and Gynecology, University Hospital and Masaryk University, Brno, Czechia

**Keywords:** gynecological cancer, microRNA, endometrium, carcinogenesis, tumor microenvironment, anticancer therapy

## Abstract

Endometrial cancer (EC) is one of the most common gynecologic cancers. In recent years, research has focused on the genetic characteristics of the tumors to detail their prognosis and tailor therapy. In the case of EC, genetic mutations have been shown to underlie their formation. It is very important to know the mechanisms of EC formation related to mutations induced by estrogen, among other things. Noncoding RNAs (ncRNAs), composed of nucleotide transcripts with very low protein-coding capacity, are proving to be important. Their expression patterns in many malignancies can inhibit tumor formation and progression. They also regulate protein coding at the epigenetic, transcriptional, and posttranscriptional levels. MicroRNAs (miRNAs), several varieties of which are associated with normal endometrium as well as its tumor, also play a particularly important role in gene expression. MiRNAs and long noncoding RNAs (lncRNAs) affect many pathways in EC tissues and play important roles in cancer development, invasion, and metastasis, as well as resistance to anticancer drugs through mechanisms such as suppression of apoptosis and progression of cancer stem cells. It is also worth noting that miRNAs are highly precise, sensitive, and robust, making them potential markers for diagnosing gynecologic cancers and their progression. Unfortunately, as the incidence of EC increases, treatment becomes challenging and is limited to invasive tools. The prospect of using microRNAs as potential candidates for diagnostic and therapeutic use in EC seems promising. Exosomes are extracellular vesicles that are released from many types of cells, including cancer cells. They contain proteins, DNA, and various types of RNA, such as miRNAs. The noncoding RNA components of exosomes vary widely, depending on the physiology of the tumor tissue and the cells from which they originate. Exosomes contain both DNA and RNA and have communication functions between cells. Exosomal miRNAs mediate communication between EC cells, tumor-associated fibroblasts (CAFs), and tumor-associated macrophages (TAMs) and play a key role in tumor cell proliferation and tumor microenvironment formation. Oncogenes carried by tumor exosomes induce malignant transformation of target cells. During the synthesis of exosomes, various factors, such as genetic and proteomic data are upregulated. Thus, they are considered an interesting therapeutic target for the diagnosis and prognosis of endometrial cancer by analyzing biomarkers contained in exosomes. Expression of miRNAs, particularly miR-15a-5p, was elevated in exosomes derived from the plasma of EC patients. This may suggest the important utility of this biomarker in the diagnosis of EC. In recent years, researchers have become interested in the topic of prognostic markers for EC, as there are still too few identified markers to support the limited treatment of endometrial cancer. Further research into the effects of ncRNAs and exosomes on EC may allow for cancer treatment breakthroughs.

## 1 Introduction

**Figure 1:**
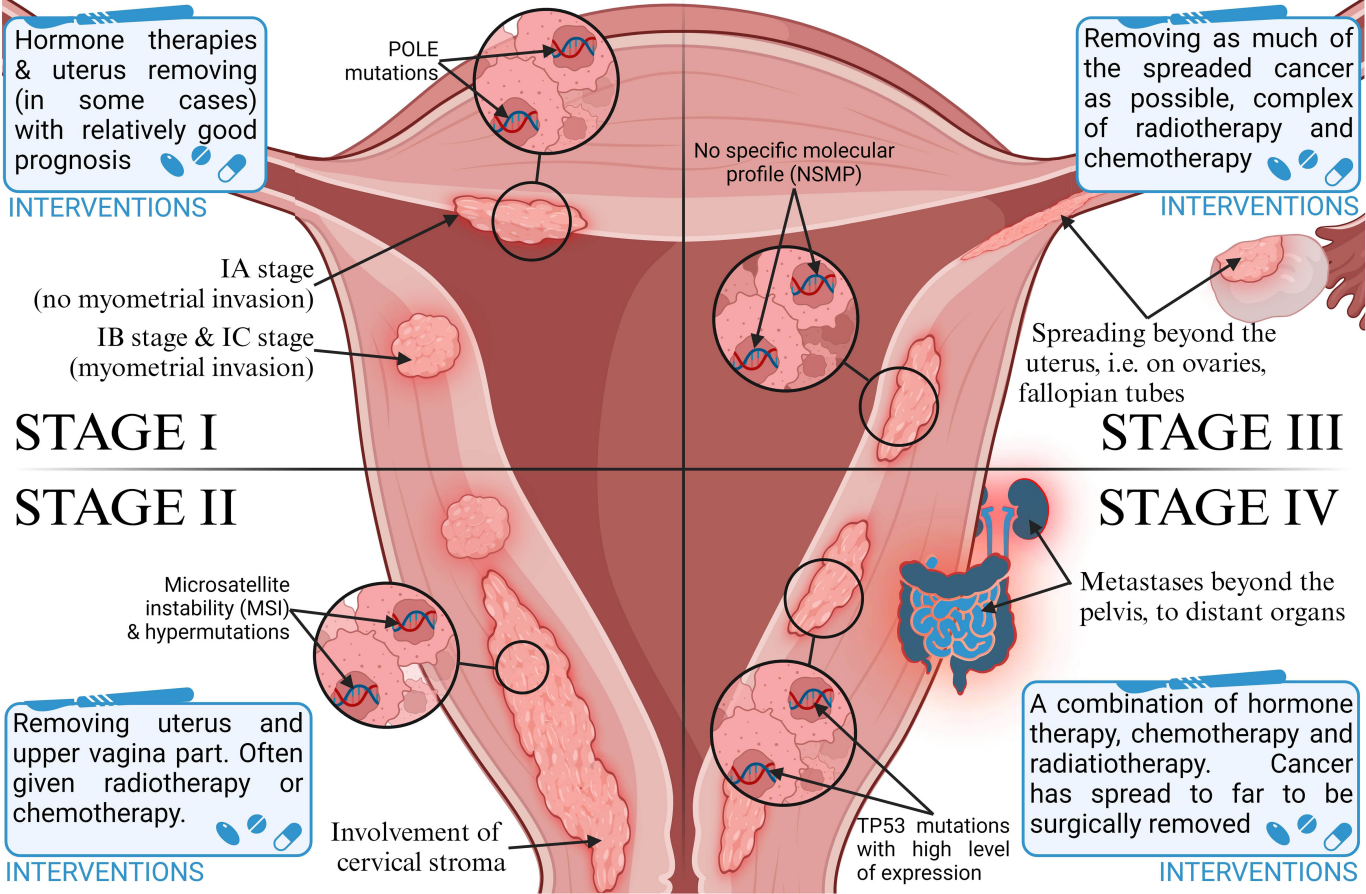
Classification of stages of endometrial cancer progression and therapeutic intervention strategies.

Endometrial cancer (EC) is one of the most common types of malignancies in women worldwide, and also the most common gynecologic cancer. According to Sung et al. (1), there were an estimated 420,000 new cases in 2021, including 97,000 deaths. In Europe, its incidence in 2021 was about 79 cases per 100,000 women (2). According to recent reports, 66,000 new cases and more than 13,000 deaths from endometrial cancer were detected in the USA in 2023, 83% of which were endometrial cancers (3). This type of cancer is divided, according to Bokhmnan’s dualistic theory, into two main clinicopathological and molecular types. Type I is endometrial adenocarcinoma, and type II includes nonendometrial subtypes such as serous, clear cell, or undifferentiated carcinoma (4). Despite early diagnosis in most patients, differences in the characteristics and histopathological features of the disease affect the prognosis as well as the recommended treatment. It also depends on the stage of the tumor (5). The most commonly used FIGO classification assigns cancer progression to four stages (6), shown in **Figure 1**. In recent years, research has focused on the genetic characterization of cancers to better determine their prognosis and personalize therapy. EC is one of the major cancers in which genetic mutations and epigenetic modifications have been found to underlie their development due to the increasing prevalence of the disease in the population (2). After a systematic review of EC prognostic markers, it was concluded that the lack of identification of specific markers is one of the main reasons for the absence of more clinical trials on EC (7). There is an urgent need to develop alternative biomarkers of EC in the blood to enable timely diagnosis for clinical use. Emerging blood biomarker tests are showing promise in cancer diagnosis, focusing on genes, proteins, and metabolites (8). Metabolic biomarkers, in particular, seem very promising due to a better picture of the genotype than gene and protein markers (9). However, current metabolite biomarkers in blood remain limited, and a panel of metabolic biomarkers needs to be constructed based on advanced analytical techniques (10). One promising marker may be phosphatase and tensin homolog deleted on chromosome ten (PTEN). It is a tumor growth suppressor that affects growth regulation and cell survival (11). Eritja et al. (12) conducted research on the mechanisms by which PTEN deficiency affects the development of endometrial cancer. Thus, it can be used as an immunohistochemical marker of EC. It has been suggested that cancer stem cells play an important role in the development of endometrial cancer because their presence is associated with resistance to chemotherapy. Various markers have been identified on the surface of these cells, such as CD40, CD44, or CD133, and their expression explains the increased carcinogenicity. However, research in this area is being further pursued, as there is a lack of strong evidence indicating their use as universal markers of EC stem cells (13).

Noncoding RNAs (ncRNAs) consist of nucleotide transcripts with very little or no ability to code for proteins. Their expression patterns in many malignancies include changes that can promote or inhibit tumor formation and cancer progression. They may also regulate organic protein-coding phenomena at the epigenetic, transcriptional, posttranscriptional, and alternative levels (2). NcRNAs play a key role not only in cellular processes but also in the pathogenesis of human diseases. They also include long noncoding RNAs (lncRNAs), microRNAs (miRNAs), and circular RNAs (circRNAs). Dysregulation of the expression of various ncRNAs and the associated competing endogenous RNA network has been found to be involved in the genesis and development of various malignancies, including EC (14). Considerable interest has been aroused by the recently discovered group of lncRNAs, which have more than 200 nucleotides. Due to their significant presence in the human genome, as well as their tissue-specific expression patterns and importance in the context of physiological and pathological processes, their length allows them to form three-dimensional structures, presumably to determine specific interactions of lncRNAs with biomolecules such as transcription factors, histones, or other chromatin-modifying proteins (15). Another of the ncRNA representatives is miRNAs. This is a specialized class of noncoding RNAs with a small size of 20 to 25 nucleotides. They regulate gene expression as well as various biological processes, such as tumor progression or self-renewal (16). The development of endometrial receptivity in embryo implantation, or endometriosis, and EC as miRNA-related factors are important gene regulators in different parts of the human body. Key species differences in endometrial remodeling make it important to remember that discoveries made in mice do not automatically apply to humans. However, the sensitivity of the endometrium in humans is quite similar to mice. There are 29 miRNAs in humans and 15 miRNAs in mice. In particular, ncRNA molecules regulating Wnt signaling were revealed, as well as those in the let-7, miR-23, miR-200, and miR-183 groups. However, future studies require further investigation into the clinical significance of these miRNAs (17). Some tumor suppressors present in mice, such as phosphatase and tensin homologs, have been well studied in ECs, while another endoribonuclease responsible for miRNA genesis (DICER1) has not been investigated. These molecules were thought to play a key role in the development and progression of EC (18).

Exosomes are round, bilayered extracellular vesicles released from many cell types, both by normal and diseased cells. Notably, cancer cells release more exosomes compared to healthy cells (19). Tumor-derived exosomes contribute to proliferation, inflammation, drug resistance, metastasis, tumor formation, and immune response. They also contain bioactive compounds such as proteins, single-stranded DNA, and genomic DNA, as well as various types of RNAs, lncRNAs, and miRNAs. Exosomal miRNAs modulate the expression of oncogenes and activate suppressors in cancer cells. Their alterations also act as a critical modulator of carcinogenesis. Moreover, oncogenes carried by circulating tumor exosomes induce malignant transformation of target cells over long distances (20). By transporting proteins, lipids, and miRNAs, exosomes show great potential to play important roles in intercellular communication. They may have significant use as tumor markers. The delivery of exosomes and exosomal miRNA has been shown to inhibit tumor progression in mice, which may signify a promising research direction in the context of human cancer (21).

## 2 Endometrial cancer—epidemiology

**Figure 2:**
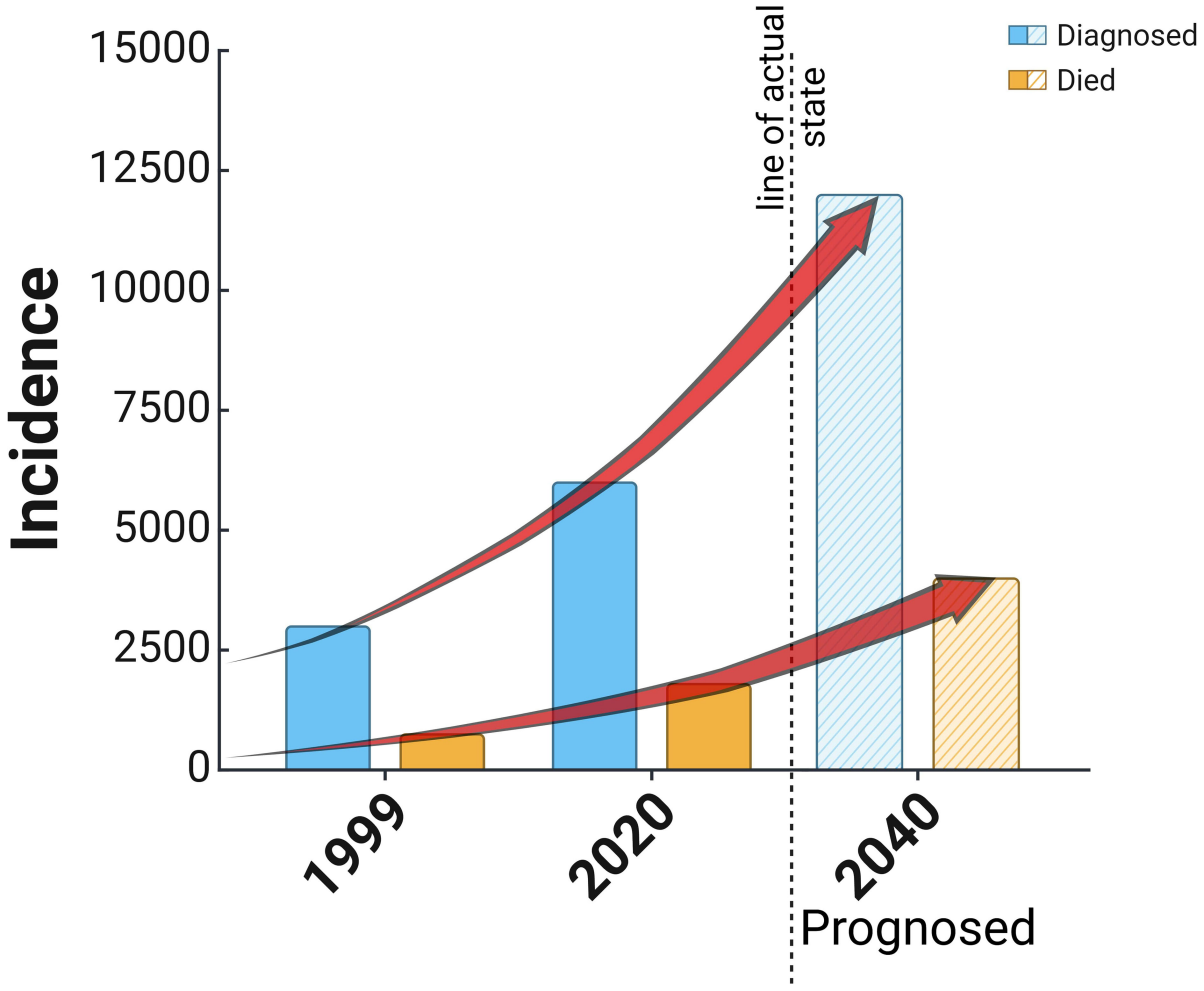
Reported cases of diagnosis and death in the cause of endometrial cancer in Poland since 1999 and a prognosis based on the actual trend of increasing cases of the disease.

EC, or cancer of the corpus uteri, is developed in the mucous membrane of the corpus uteri. Women aged 60 or older are affected, with mortality peak in 70 to 80 years. EC is the most common gynecological cancer in developed countries (22). In recent years, the incidence of endometrial cancer has been gradually increasing due to improved socioeconomic levels, accelerated aging of the population, and an increase in obesity and metabolic syndrome (1). According to the WHO, the worldwide incidence of endometrial cancer is 420,368 women, while the mortality rate is 97,723 women, which places it 15th among the most common cancers worldwide (23). There were circa 100,000 newly diagnosed cases in Europe every year, with an incidence of 13–20/100,000 and a low mortality rate of 2–2.7/100,000 women (24, 25). EC is the fourth (after breast, lung, and large intestine) cancer in women in Poland; it is diagnosed in circa 6,000 women, and each year more than 1,500 patients die. The morbidity and mortality ratio is continually rising, as shown in **Figure 2** (26), which occurs for unknown reasons.

There are several recognized EC risk factors, among them increased body mass index (BMI). For BMI 22–27.2, the increased incidence is +21%; for BMI 27.5–29.5, it is +43%; and for BMI more than 30, it is +273%. The remaining factors are age after 50–60, hypertension, hyperinsulinemia (or insulin resistance), and prolonged exposure to estrogen (often in infertility, nulliparity, polycystic ovarian syndrome, tamoxifen intake, early menarche, and late last menstruation). In very rare congenital syndromes such as Lynch or Cowden, the risk of EC is higher. The EC risk is lower in women having children, physical activity, a high-fat diet preventing obesity, contraceptive therapy (estrogens and androgens), intrauterine devices, and coffee (22, 25, 27).

There were initially two subtypes of endometrial cancer based on histopathology. The endometroid adenocarcinoma was classified as type I, and all other cancers (serous, clear cell, carcinosarcoma, and undifferentiated) were type II (4). The first type was related to good and the second type with poor prognosis. In the 1st type, there was an atypical endometrium hyperplasia, which is considered a precancerous state—in which 25% of cancer could develop. There were no precancerous states in the second type of cancer. Tumors are graded according to the International Federation of Gynecology and Obstetrics criteria as grades 1 (G1) and 2 (G2) as low grade and grade 3 (G3) as high grade (28). Several factors have been identified as recurrent risk: G3, at least 50% of myometrium invasion, histological pattern, lymphovascular space invasion, lymph node metastases, and tumors bigger than 2 cm (25).

Classification based on histopathology has been replaced by a system based on molecular phenotypes. More than 90% of ECs are sporadic; the rest are hereditary, most of them related to nonpolyposis colorectal cancer syndrome (HNPCC) or Lynch syndrome. The HNPCC patients have an increased EC developing risk of circa 10-fold, except for the possibility of colon or ovarian cancer. These are usually microsatellite-unstable tumors observed in younger women (29).

## 3 Current preclinical and clinical research on endometrial cancer

EC preclinical studies play a pivotal role in providing general information about the impact of the tested drug, dose, or therapy on a living organism. Several drugs have been produced for cancer research using cell lines, but some drug’s effectiveness in *in vitro* models is disproportionate to that observed in the human body. Commonly used immortalized cancer cell lines are cost-effective and convenient to work with; however, they are genetically unstable and less representative of the clinical phenotype observed in patients. Consequently, the EC preclinical studies are based on, e.g., animal models. The *in vivo* model makes a great opportunity for proper research, often based on invasive procedures. They allow research to be conducted in a way that would be impossible in humans but in accordance with ethical standards (30).

One of the examples of the anima model usage may be patient-derived xenograft (PDX) models. PDX are models of cancer where the tissue or cells from a patient’s tumor are implanted into an immunodeficient or humanized mouse. This strategy simulates human tumor biology allowing for natural cancer progression, and enables the most translational research model for evaluating efficacy. Furthermore, PDX models are applied to various cancers, such as endometrial, breast, colon pancreas, stomach, bladder, lung, cervix, and kidney (31). Imai et al. (32) investigated the presence or absence of the molecular properties of endometrial carcinomas in PDXs passaged up to eight times. It has been reported that established PDXs of endometrioid carcinomas maintained their histopathological characteristics; however, those of carcinosarcomas predominantly consisted of sarcomatous components in comparison to the parental tumors. Alterations in the proportion of cells with immunohistochemical staining for estrogen receptor, PTEN, paired box 2 (PAX2), and paired box 8 (PAX8) (32) are frequently observed. PTEN is the most frequently altered gene in human EC. Genomic analyses of ECs have revealed many recurrent genetic changes as well as the deregulation of signaling pathways. One of the biggest drawbacks of the current hybrid *ex vivo*/*in vivo* model of carcinogenesis is the lack of an immune response and tissue-specific physiological microenvironment. Most previous studies have focused on histological features but not on genomic, transcriptomic, and proteomic features regarding the similarity between induced mouse uterine tumors and human ECs (33). The molecular mechanism of pathogenesis is not yet sufficiently described. Hybrid *ex vivo*/*in vivo* modeling of EC may accelerate the understanding of EC (34).

Paired box (PAX) is a family of gene factors coding for tissue-specific transcription factors that play key roles in cell fate, early patterning, and organogenesis (35). Using this approach, Wang et al. (36) investigated the function of PAX2 on endometrial cancer both *in vitro* and *in vivo*. The results revealed that PAX2 remarkably enhanced proliferation and invasiveness. Moreover, PAX2 impacts the expression of cyclin-dependent kinase 1 (CDK1), which plays pivotal roles in the cell cycle pathway (35). PAX8 belongs to the paired-box gene family, which plays an important role in the organogenesis of different body organ systems. PAX8 plays a significant function in tumor metastasis, embryo development, central nervous system, angiogenesis, and immune regulation (37). Another example of using a mouse as a research model is the transforming growth factor beta (TGF-β). Protein signaling plays an important role in the development of endometrial cancer. PTEN depletion is one of the important factors in developing endometrial cancer in mice. Monsivais et al. (38) introduced and analyzed a mouse model with conditional inactivation of activin receptor-like kinase 5 (ALK5) in the uterus using progesterone receptor—the mice developed endometrial adenocarcinoma with metastases to the lungs. In this study, the authors postulated that TGF-β signaling through TGF-β receptor/ALK5 is responsible for tumor suppression and endometrial regeneration (38). Ruiz−Mitjana et al. (39) addressed the role of the extracellular matrix in the cellular responses to TGF-β. They showed that in the absence of an extracellular matrix, TGF-β-treated endometrial epithelial cells display features of epithelial-to-mesenchymal transition and also used mice housed in a barrier facility and pathogen-free procedures in all mouse rooms (37). The enhancer of zeste homolog 2 (EZH2) is also contributed and studied in endometrial cancer. In this study, mice harboring a uterine deletion of both Ezh2 and Pten showed reduced tumors in the early stage of cancer development. Unfortunately, the authors observed increased inflammatory reactions (40).

Furthermore, small-animal imaging in EC has increased over the last decade. Preclinical imaging enables *in vivo* observation of long-lasting therapeutic responses and monitoring of tumor growth and metastatic spread. Consequently, preclinical imaging provides imaging biomarkers for prediction and evaluation of treatment (41) Moreover, previous preclinical EC studies focused on caliper size measurements of less relevant subcutaneous models using cell lines (42). Pelvic magnetic resonance imaging (MRI) and whole-body positron emission tomography-computed tomography (PET-CT) play an essential role in primary diagnosis and in detecting recurrent EC disease in patients. Using this approach Espedal et al. (43) developed EC organoid-based orthotopic mouse xenograft models (O-PDX), in which they imitated tumor tissue, namely the histopathologic architecture and protein biomarker expression (43) Computed tomography (CT) is used to find lymph node metastases in EC patients. It has been reported that in preclinical studies, CT has been used to detect both local and advanced diseases. We applied the orthotopic EC xenograft using the estrogen-dependent Ishikawa cell line. In an estrogen-controlled orthotopic model of EC using contrast-enhanced CT (CE-CT), image-derived tumor volume was found to be positively correlated to tumor net weight at necroscopy. SPECT is a type of imaging test that uses a radioactive substance and a special camera to create 3D pictures. This test is also known as single-photon emission computerized tomography. While many imaging tests show what the internal organs look like, a SPECT scan can show how well the organs are working. In preclinical EC models, SPECT takes part in monitoring the treatment efficacy of two different strains of oncolytic viruses (Copenhagen and Wyeth vaccinia virus) in subcutaneous cell-line xenografts (44).

Another example is organoid-initiated precision cancer models (OPCMs). These models enable the functional studies of genetic alterations in tumorigenesis and create precision models for preclinical drug testing. The above model was used, and it was shown that the mutations in phosphatidylinositol-4,5-bisphosphate 3-kinase catalytic subunit alpha (Pik3ca) and phosphoinositide-3-kinase regulatory subunit 1 (Pik3r1) cooperate with PTEN loss to promote endometrial adenocarcinoma in mice. On the other hand, the Kras G12D mutation induces endometrial squamous cell carcinoma (45).

in addition to the mentioned technologies, the most used prognostic and diagnosis tools for cancer are molecular markers, such as proteins or gene mutations. There is a wide list of commonly used markers, the quantitative or qualitative identification of which gives a limited insight into the diagnosis and progression of the disease, but their importance still remains very significant. The importance of many of the factors involved in the pathogenesis and development of cancer is described in detail below.

### 3.1 Estrogen receptors

Also noteworthy in preclinical studies is the role of estrogen, which can affect the endometrium by interacting with estrogen receptors (ER) to induce endometrial proliferation during the proliferative phase and progesterone receptor (PR) synthesis, which prepares the endometrium for the secretory phase. Knockout studies in mice have shown that the expression of ER, including ERα, ERβ, and G-protein-coupled estrogen receptor (GPER) in the endometrium, is crucial for the normal function of the female reproductive system (46). The possible role of GPER in the regulation of reproductive function was further confirmed by Prossnitz et al. (47), demonstrating that GPER mRNA expression in the endometrial and mammary gland epithelium was regulated in an estrous cycle-dependent manner. In addition, gonadotropic hormones affected GPER protein expression in the granulosa and sheath cells of hamster ovaries. The above results may suggest that GPER may be involved in the regulation of pre-antral follicle development (48).

Abnormal ER expression can cause many diseases, such as endometriosis, endometrial hyperplasia (EH), and endometrial cancer (EC). ERα promotes endometrial cell proliferation and is strongly associated with an increased risk of EC, while ERβ has the opposite effect on ERα function. GPER is strongly expressed in abnormal EH, but its expression in patients with EC remains paradoxical (49). Successful treatment of endometrial-related diseases depends on understanding the physiological function of the ER (46).

### 3.2 Molecular markers

#### 3.2.1 CD40

With innovative therapeutic methods and the integration of molecular analysis, the treatment of endometrial cancer is undergoing a profound transformation these days. In the past few decades, treatment methods have been limited to surgical resection, chemotherapy, radiation therapy, and hormonal therapy. However, in recent years, therapy based on specific biomarkers has begun to be practiced (50). To investigate tumorigenesis more broadly, it is also worth focusing on proteins that may have an important role in carcinogenesis. This will allow effective blocking of signals necessary for EC cell progression (51). CD40 is a protein that plays an important immunoregulatory role in tumor regression. It has a dual function, both antitumor and protumor. This is due to the expression of proinflammatory and anti-inflammatory cytokines, which is dependent on the strength of CD40 stimulation (52). Some of the pro- and anti-inflammatory mediators, such as vascular endothelial growth factor, help in angiogenesis by producing capillaries. However, they are also often channels for the metastasis of cancer cells (53). It is also included in the tumor necrosis factor (TNF) receptor family, which, when activated, can mediate tumor regression. However, despite the great potential of CD40 agonists, there are few studies on their therapeutic effects on cancers of the reproductive system (54). CD40 activation can significantly affect the tumor microenvironment, independent of innate immune sensors. CD40 knockout mice showed impaired stimulation of T cells to switch classes or form reproductive centers (55).

#### 3.2.2 IL-11

IL-11 belongs to the IL-6 family of cytokines that promote Th17 differentiation in both mice and humans. Th17 cells are proinflammatory cells that secrete cytokines and provide resistance to extracellular pathogens, including defense against infections (56). However, IL-6 alone does not induce Th17 differentiation, unlike IL-11, which induces Th17 cell differentiation and expansion. In the context of signaling pathways in the tumor microenvironment, cytokines such as IL-6 and IL-11 can regulate proliferation, survival, differentiation, or cell death. They can also induce an antitumor response or induce cell transformation and malignancy (57). According to Wang et al. (51), YAP is a transducer protein involved in endometrial cancer progression through upregulation of IL-6 and IL-11. YAP is increased in endometrial cancer cell lines and tissues to a greater extent than in endometrial stromal cells or benign tissue and is involved in endometrial cancer progression with IL-6 and IL-11 (58).

### 3.3 Programmed death receptor

A programmed death receptor (PD-1) is a negative costimulatory molecule that exerts negative effects on T cells by inhibiting cytokine production and cell proliferation through decreased cytokine expression (59). PD-1 is a cell surface molecule consisting of 288 amino acids. It is a membrane protein in the superfamily of human immunoglobulins. Its role is to suppress the acquired and innate immune response. It is found in immune cells, including activated T cells. It is worth noting that tumor-specific T cells show high expression of PD-1 (60). PD-1 exhibits a dualistic nature, encompassing both beneficial and detrimental effects. It reduces ineffective or harmful immune responses and maintains immune tolerance. However, PD-1 activation may contribute to the development of malignant cells by impeding protective immune responses (50). This type of protein has two types of ligands: programmed cell death ligand 1 (PD-L1) and programmed cell death ligand 2 (PD-L2). They show different expression patterns, are located on chromosome 9p24.1, and encode the CD274 and PDCD1LG2 genes, respectively (61).

PD-L1 is usually detected in macrophages, activated T cells, NK cells, and some epithelial cells in the presence of inflammatory stimuli. In addition, tumor cells use PD-L1 expression to evade antitumor responses (62). The induction of protein kinase D isoform 2 (PKD2) by interferon-gamma (IFN-γ) plays an important role in the regulation of PD-L1. Inhibition of PKD2 activity suppresses PD-L1 expression, increasing the efficacy of the antitumor immune response (63). PD-L1 thus acts as a tumor growth promoter, engaging specific receptors and initiating signaling pathways that encourage cell proliferation and survival. This provides evidence that PD-L1’s involvement in cancer progression is significant. The PD-L1 pathway plays a role in facilitating tumor growth (64). The inclusion of PD-1/PD-L1 inhibitors in anticancer therapy seems promising. However, it is important to remember that the human immune system may create some roadblocks to its effectiveness, which may result in the opposite of the intended effect (65).

### 3.4 Chimeric antigen receptor for the T cells

Transfusion, which involves the infusion of lymphocytes to induce an antitumor response, also deserves special attention. This is a rapidly developing field, from the immuno-oncological form in preclinical studies to the chimeric antigen receptor for the T cells (CAR-T), which has found application in the treatment of leukemia and lymphoma, among others (66). CAR, or chimeric antigen receptor T-cell therapy, involves modifying a patient’s own T cells by attaching special receptors called chimeric antigen receptors (CAR) to them. CARs recognize and bind to specific antigens on the surface of tumor cells (67). Therapy with the abovementioned cells relies on the selection of a target antigen. This specific antigen must have higher expression against cancer cells and minimal expression in healthy tissues. This allows T cells to distinguish between healthy and diseased cells. In summary, this therapy aims to genetically program a patient’s T cells to target cancer cells in order to kill them (68). CAR-T-cell therapy has shown great promise for treating certain hematologic cancers. However, researchers continue to face obstacles related to the safety and efficacy of this type of therapy (69). In recent years, some miRNAs have also been isolated, including miR-34-5p, which are involved in the regulation of cancer cell proliferation, apoptosis, and metastasis. Novel CAR^miR^ cells have been developed that can serve as a cell-based proof-of-concept therapeutic for *in situ* production and delivery of miRNAs with therapeutic applications. This was developed by cloning a CAR molecule with interleukin-13 (IL-13 E12Y), which was modified. It exhibits high affinity binding to tumor cells. When CAR is stimulated with cancer antigen, upregulation and export of miR-34a-5p exosomes is induced. Exosomes isolated from CAR^miR^ cells have shown increased toxicity to cancer cells (70). MiRNA-34a is a tumor suppressor microRNA that is absent in many cancer stem cells (CSCs) and advanced cancers. It has been identified as a target of p53, and studies have shown that miR-34a is an essential mediator of p53 function and a potent tumor suppressor (71). MiR-34a inhibits tumor growth and tumorigenesis by inhibiting cell cycle and metastasis, among other things. Moreover, it induces tumor-suppressive processes such as apoptosis and cellular aging (72). The new CARmiR cells, due to their enhanced cytotoxic activity, are predicted, compared to CAR cells, to be an effective tool with high clinical potential. This may contribute to significant advances in CAR-T therapy for cancer treatment (70).

### 3.5 Cancer stem cell markers

CSCs also play an important role in cancer progression. Cancer stem cells share many similarities with healthy stem cells. When introduced into an animal model, they show the ability to self-renew, differentiate, and induce tumor formation. There is a specific group of markers that are often used to enrich and identify CSCs. These include CD133, CD90, and CD44, among others (73). Research shows that prominin glycoprotein 1 (CD133) is essential for metastasis and proliferation of cancer cells. The proliferative capacity of CD133^+^ cells is not clearly defined. However, it is speculated that they may have a reservoir function for producing more cells capable of metastasis. CD133 has also been found to have a strong effect on the growth and resistance of CSCs to chemotherapy (74). CD90, also known as Thy-1, belongs to the glycoproteins anchored by glycosylphosphatidylinositol (GPI). Studies show that it belongs to CSC markers in the context of lung cancer (75). A study by Cao et al. (76) shows that spheroid cells showed increased expression of several markers, including CD133 and CD90. Thus, they may be used as a promising treatment option for endometrial cancer. There are also ongoing studies on tumor heterotransplant cell lines to demonstrate the ability of CD44^+^ to induce tumor formation in NOD/SCID mice (77). Studies (36) also show that CD44 is a marker of CSCs and a regulator of tumor self-renewal, initiation, and metastasis. CD44 can therefore be used to isolate or enrich CSCs through fluorescence-activated sorting of patient cells, tumor tissue heterotransplantation, or cell culture.

## 4 Signaling pathways involved in induction, progression, and metastasis of endometrial cancer

### 4.1 Janus kinase/signal transducer and activator of transcription 3

**Figure 3:**
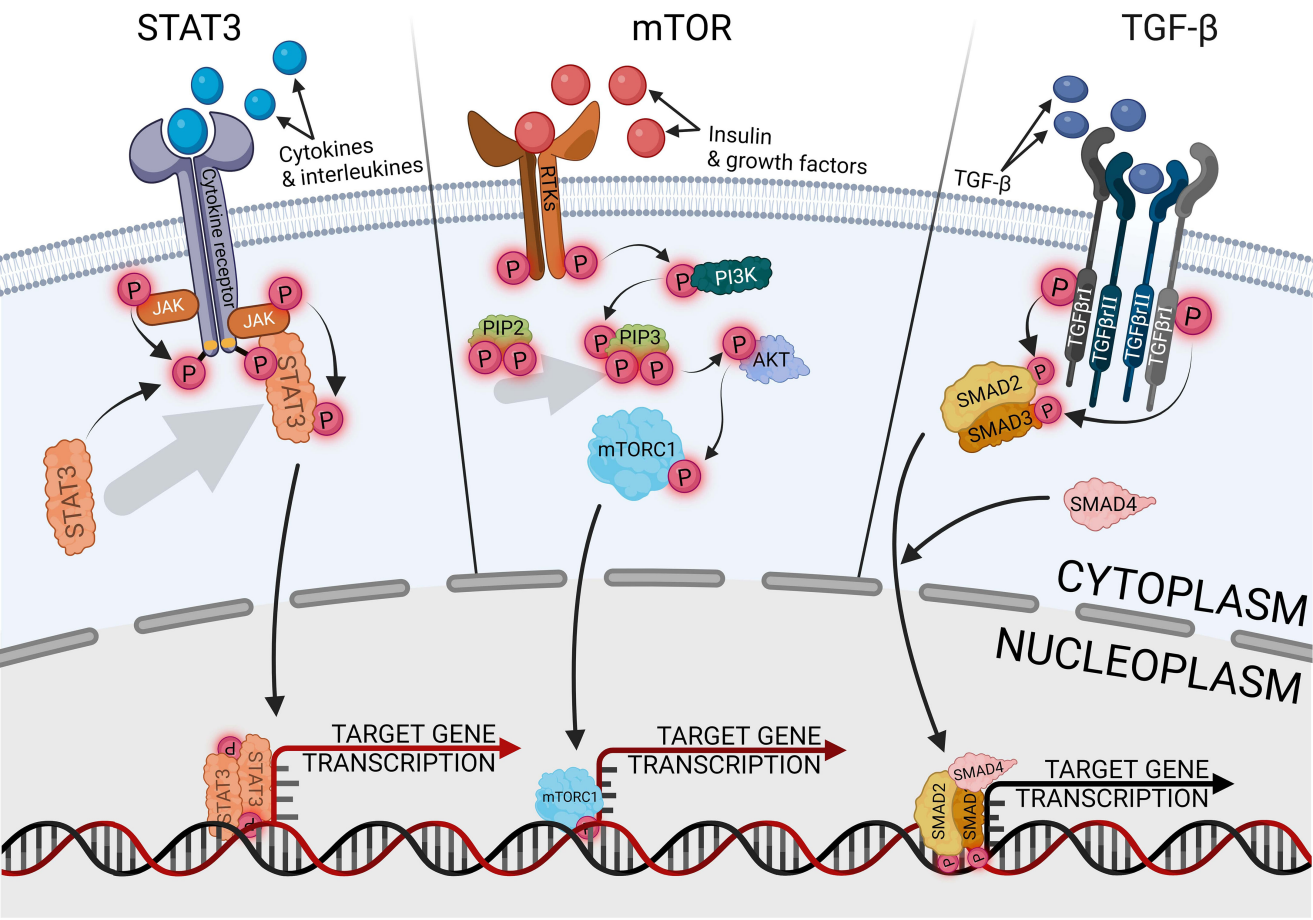
Canonical ways of activating signaling pathways of STAT3, mTOR and TGF- β.

The signal transducer and activator of transcription 3 (STAT3) is the most carcinogenic prominent protein in the whole signal transducer and activator of transcription (STAT) protein family. It is engaged in activating and controlling a wide variety of cancer hallmarks, such as proliferation, cell stemness, metastasis, angiogenesis, immunosuppression, inflammation, and reprogramming metabolism (78). Another STAT3 role is cell cycle regulated, especially G1- to S-phase transition and G2/M-phase checkpoint via cyclins p21 and p27 (79). Therefore, STAT3 has become a promising marker and potential therapeutic target in various types of cancers, such as lung, prostate, pancreatic, breast, and endometrial cancer (80–84). The canonical pathway of STAT3 activation is induced by the binding of an extracellular ligand, such as cytokines and growth factors, with a receptor complex composed of the cell membrane receptor, Janus kinase (JAK), and STAT3, as shown in **Figure 3**. Ligand–receptor interaction provides STAT3 phosphorylation and separation from the complex via JAK signaling (85). The most well-known traditional activator of the JAK/STAT3 pathway is interleukin-6 (IL-6), indicated as the most significant ligand with carcinogenic potential, which also contributed to endometrial cancer development (86). Elevated levels of IL-6 are strictly related to obesity and oxidative stress, especially in EC, and are associated with a poorer prognosis (87). IL-6, as a multifunctional factor, promotes endometrial cancer, not only in relation to JAK/STAT3. Regulatory activity of IL-6 is universally reported in many tissue cancers, negatively influencing drug chemoresistance and enhancing cell proliferation (88, 89). Other signaling pathways that are regulated with IL-6 in EC are cGAS-STING, nuclear factor kappa B (NF-κB), HIF1α, VEGF, mTOR, and above all, STAT3-related pathways (51, 90, 91). Hyperactivation of STAT3, which is especially linked with tumors, can occur through the following mechanisms: extended activity of receptors for pro-oncogenic ligands; excessive cytokines or growth factor stimulation; enhanced activity of cytoplasmic nonreceptor tyrosine kinases, like JAKs or Abelson kinases, directly or indirectly related to STAT3 activity. The fourth mechanism is a loss of negative JAK/STAT3 regulation (92). In physiological conditions, the negative pathway control has proceeded with protein inhibitors of activated STAT (PIAS) and suppressors of cytokine signaling (SOCS) protein families. The SOCS are shielding STAT- and JAK-binding sites, preventing the activation of subsequent stages of the activation cascade, while the PIAS are blocking active STAT dimers from binding to DNA (93). The universality and ubiquity of STAT3 make it difficult to determine at what stage of cancer development it plays the greatest role. In the early stages of cancer, the STAT3 pathways participate in several tissues, which are linked with cancer growth upregulating cell stemness and proliferation (94, 95). In cases of endometrial cancer, STAT3 inhibition reduces primary cell viability, proliferation, and invasion, impairing not only tumor development but also metastasis (11, 96, 97). Migration and invasion properties of endometrial cancer cells are provided via upregulating, among others, cyclooxygenase-2 (COX2) and matrix metalloproteinase 2 (MMP2) (86, 98). Activated STAT3 directly binds to a lncRNA promoter, promoting its expression and having the ability to form STAT3/lncRNA/miRNA-positive feedback loops (10). Among others, STAT3 promotes CASC9 expression, essential lncRNA in glioma cells, which upregulates the expression of STAT3 via sponging miR-519d, generating a positive feedback loop of STAT3/CASC9/miR-519d (99). LncRNA is involved, among others, in initiating a prometastatic endometrial cancer phenotype; nuclear paraspeckle assembly transcript 1 (NEAT1)-mediated miR-361 sponging activates the STAT3 axis. Modulating the Stat3 axis also engages the myocyte enhancer factor-2 (MEF2), Rho-associated protein kinase 1 (ROCK 1), Wnt family member 7A (WNT7A), and Karyopherin subunit alpha 4 (KPNA4), thus suppressing only one of the factors will inhibit the prometastatic cascade (100).

In endometrial diseases, the JAK/STAT3 pathway is especially triggered with leptin, a protein hormone related to obesity and predominantly expressed by adipocytes (101). The deregulated JAK/STAT3 pathway is a common factor in endometrial cancer and diabetes. Diabetes constitutes a significant risk factor for endometrial cancer induction and development; conversely, a diabetes prevention diet may decrease the occurrence and development of endometrial cancer (102). The symptoms of diabetes may be modulated by regulating insulin and insulin growth factor (IGF) levels, which activate the JAK/STAT3 pathway (18). That effect may be reduced by using antidiabetic drugs, like metformin, which inhibit IGF activity (103), but there is a poor chance of inhibiting or blocking the development of cancer using metformin (104). The effects of metformin may only reduce the upregulating effects of high glucose conditions on JAK/STAT3 levels (105).

### 4.2 Transforming growth factor-β

TGF-β is a wide family of secreted cytokines, demonstrating conservativeness in the whole animal kingdom and appearing in the early days of multicellular evolution. It fulfills universal signaling functions that provide tissue- or cell-specific control of differentiation, proliferation, and metabolism across the full spectrum of embryonic and adult development (106). Through the developmental processes, TGF-β pathways are responsible for the regulation of cell proliferation and apoptosis, especially in terms of endometrial epithelial cells (107). The canonical TGF-β uptake proceeds with recipient cells’ receptor kinases, mediating phosphorylation of the SMAD protein family, activating the cascade, and manipulating the expression of target genes, as shown in **Figure 3** (108, 109). The signaling pathway is important in in tumor development and metastasis of gynecological cancers, but it also plays a dual role, activating or suppressing carcinogenesis depending on the tissue (110). One of the family members, bone morphogenetic protein (BMP), has a tumor-promoting effect on ovarian and endometrial cancer, whereas it fulfills a protective role on uterine cervical cancer (111). In endometrial cancer, enhanced activity of the TGF-β/Smad pathway is correlated with tumor progression, metastasis, and recurrence (112), and its implication in the malignant phenotype of endometrial tumors also promotes cell stemness (113, 114). TGF-β enhances the malignancy of endometrial cancer by disturbing the epithelial permeability barrier, cell migration, and mitochondrial metabolism (115). The effect of endometrial cancer development is additionally increased by the immunosuppressive effect of TGF-β, disrupting the functionality of natural killer cells and T cells (116, 117). The regulation of the TGF-β/Smad pathway occurs variously and is modulated according to the expression of other proteins, especially in cancers with inactive TGF-β expression. The activity of the pathway is counter-proportionally correlated with cancer-related proteins, like flotillin 2 (FLOT2) or early growth response 2 (EGR2) (118, 119). Targeting the miR-650/SMAD7 axis via lncRNA MCTP1-AS1 suppresses cell proliferation, migration, invasion, and epithelial–mesenchymal transition in endometrial cancer (120). The result of stopping that axis is downregulation of the TGF-β activity, demonstrating therapeutic potential. In other research, inhibiting lncRNA AFAP1-AS1 negatively regulated the TGF-β/Smad axis, influencing proliferation, migration, and apoptosis via miR-424-5p. The AFAP1-AS1 effect was demonstrated on an endometriosis model, but potentially inhibiting this signaling pathway may also support the inhibition of endometrial cancer development. Due to endometrial cancer related to metabolic syndrome, the pro-oncogenic effect of TGF-β/Smad is enhanced by lysophosphatidylcholine acyltransferases (LPCATs), a regulator of intracellular lipid metabolism, which promotes cell stemness and metastasis (121). The opposite effect can be achieved by using metformin, a well-known antidiabetic agent, which has a neoplastic effect by silencing the activity of the pathway (39). Protein kinase C α (PKCα) activity affects the TGF-β receptor 1 (TGF-βR1), which positively influences the TGF-β ability to induce SMAD phosphorylation and cell cycle arrest (122). The process is dependent on extracellular signal-regulated kinase 2 (ERK2) and runt-related transcription factor 2 (Runx2), and the knockdown of either of the genes immediately suppresses tumorigenesis (123).

### 4.3 Mammalian target of rapamycin

The mammalian target of rapamycin (mTOR) plays a central role in endometrial behavior and fertility, being involved in estrogen signal transduction. Dysregulation of the mTOR pathway is critical in the induction and progression of endometrial diseases, including cancer (124). Additionally, in various tumors, mTOR signaling interacts and loops with another axis, like the WNT/β-catenin pathway, which may activate mTOR signaling, while mTOR signaling may suppress the WNT/β-catenin pathway (125). Both of the above are the key regulators of endometrial cancer progression (126). The interaction of the pathways may be useful for indicating tumor grading. Identification of WNT in the cell membrane may exclude the third grade of neoplasms, whereas the presence of cytoplasmic WNT and nuclear mTOR may indicate the third grade of neoplasms (127). The canonical pathway of mTOR activation is preceded by phosphoinositide 3-kinase (PI3K) and protein kinase B (AKT or PKB) activity, as shown in **Figure 3**, the standard signaling pathway in tumor induction and progression, promoting cell metabolism, proliferation, migration, and angiogenesis (128). Pro-oncogenic properties of the pathway can be suppressed at many levels, such as blocking PI3K with p53, substituting AKT with the MLLT11-TRIL molecule complex, or inhibiting mTOR with AMPK upregulation (129–131). Suppressing activity of the AKT/mTOR pathway with some of the factors effectively inhibits the tumor tissue activity (132, 133), but endometrial cancer therapies engaging only a single inhibiting factor are insufficiently effective as monotherapy (134); thus, using some factors enhancing the suppressing mTOR inhibitors effect significantly intensifies reducing endometrial cancer cell viability, colony formation ability, and induced apoptosis (135). The most frequently followed genetic alterations in endometrial cancer are aberrations of PTEN, which is a negative PI3K/AKT/mTOR regulator. The mutation or deletion of the gene leads to disturbed activation of the PI3K/AKT/mTOR pathway, which occurs in 60%–80% of endometrial cancer cases (136).

## 5 Dysregulated noncoding RNA profile in endometrial cancer

Genome biology has evolved beyond the traditional comprehension that DNA is merely transcribed in order to be translated into protein molecules. The majority of the RNA transcripts do not code for any proteins. Instead, they govern dozens of intracellular and intercellular processes in normal or diseased settings (137). To address some functional models for these ncRNAs, lncRNAs depict multiple roles in the modulation of chromatin structure, gene silencing, mitochondrial homeostasis, and protein recruitment via interaction with other RNAs. It appears to be rational to consider networks and pathways in ncRNAs’ biology and function to achieve a plenary viewpoint (138, 139). Through influencing the gene expression profile, diverse types of ncRNAs modify the cell phenotype. Malignancy is a major landscape in which ncRNA dysregulation is a significant determinant (140). miRNAs, circRNAs, PIWI-interacting RNAs (piRNAs), and lncRNAs play divergent roles in cancer development, invasion, and metastasis, as well as in cancer drug resilience via different mechanisms such as apoptosis suppression and CSC progression (140, 141). According to the literature, ncRNAs may be practical therapeutic objects and handy clinical markers in various cancer types (142, 143). Previous studies have also revealed the dysregulated patterns of ncRNAs in EC. Noncoding RNAs especially miRNAs and lncRNAs, affect many pathways in EC tissues, leading to EC cell viability or mortality. For example, lncRNA GAS5 overexpression causes apoptosis in EC cells, and its expression is reduced in EC tissues. GAS5 targets the miR-222-3p/p27 axis, resulting in the suppression of EC progression secondary to elevated glucose levels (144, 145). In addition, by investigating EC cell lines KLE and HEC-1-B, (146) demonstrated that miR-25-3p binds and inhibits *BTG2* gene mRNA, causing EC proliferation and motility. They also reported that lncRNA SNHG5 acts as competing endogenous RNA (ceRNA) to sponge miR-25-3p and attenuates EC progression (147). Also, the differential levels of circRNAs have been investigated in EC. (148) demonstrated that hsa-circ-000579 is upregulated in EC tissue samples and is a positive regulator of EC. According to the fact that miR-298, which targets the oncogene CTNND1, is downregulated in EC cells, this circRNA was introduced as miR-298 sponge by utilizing related assays. Thus, hsa-circ-000579/miR-298/CTNND1 axis functioning is related to unfavorable outcomes (149). In a recent study, Lai et al. (150) discovered that the lncRNA BMPR1B-AS1 is highly expressed in EC tissues compared to its normal counterparts. They also reported that this lncRNA causes aggressive behaviors in EC cells through mechanisms like cell cycle activation and promoting mesenchymal features. BMPR1B-AS1 targeted and negatively regulated miR-7-2-3p in EC cell lines, leading to DCLK1 upregulation. Due to the fact that DCLK1 promotes EC via the PI3K/Akt/NF-κB pathway, BMPR1B-AS1/miR-7-2-3p/DCLK1 axis was propounded as a machinery for EC promotion (150). CCP110 is a conserved protein essential for centrosome function, and its upregulation is associated with invasive behavior in tumor cells. In an attempt to reveal the regulation of this protein in EC cells, (151) found that CCP110 is overexpressed in EC cells, and they reported that miR−129−2−3p inhibits the translation of CCP110 mRNA as well. Moreover, their research revealed that lncRNA XIST is highly expressed in EC cells and acts as ceRNA to sponge miR−129−2−3p. Therefore, the XIST/miR−129−2−3p/CCP110 axis was suggested to be involved in EC advancement (151). Overexpressed lncRNA HEIH has been discovered to be a factor for EC cells’ resistance to paclitaxel via MAPK signaling enhancement, which interferes with HEIH function, leading to vulnerability of the EC cells exposed to paclitaxel treatment (152). LncRNA SNHG4 is also expressed at higher degrees in EC cells and is correlated with cancer cell proliferation and migration. It is believed that this effect is established proportionally through upregulation of epithelial-to-mesenchymal transition (EMT) markers and transcription factor SP-1 (153).

## 6 Exosomes and tumor biology: emphasis on noncoding RNA cargos

Tumor structure is known to be an organized system of diverse cellular and other biological components. The innumerable processes in a highly dynamic environment make it essential for the tumor tissue to be under continuous rearrangement. The main factor is the effective communication apparatus of the tumor cells with other cells and tumor microenvironment (TME) cells (154). Exosomes have been described for a couple of decades to act as interesting tools of paracrine crosstalk in TME. These cup-shaped membranous vesicles shed outside the cells have an average diameter of approximately 100 nm and are capable of carrying multiple molecular cargos, including proteins, lipid products, DNA, and coding and noncoding RNAs (155). Exosomes could either deliver the components to the neighboring cells or may be transmitted by the bloodstream to exert their effects remotely. On most occasions, the net consequence of exosomal signal transduction is to facilitate tumor progression and invasion. Many exosomal elements take part in oncogene expansion, tumor suppressor gene regulation, angiogenesis, metastasis, and drug resistance (156). Exosomes also play a role in the modulation of cancer immunity by mediating the expansion or repression of tumor-residing immune cells (157). Exosomes secreted by the cancer cells were found to carry the checkpoint protein PD-L1 and suppress the anticancer immunity exerted by cytotoxic T cells. Also, the PD-L1-containing exosomes may inhibit the maturation of dendritic cells, leading to diminished T-cell immunity (158, 159). Exosomes are considerable factors that pave the way for tumor metastasis. Their components may induce EMT via several mechanisms, like upregulation of mesenchymal markers or EMT-related signaling pathways (e.g., N-cadherin and STAT3, respectively) (159, 160). Aiming to facilitate tumor invasion, exosomes also mediate the extracellular matrix (ECM) remodeling by degradation and/or synthesis of the ECM network (161). Interestingly, tumor-derived exosomes are remarkable factors in the determination of target metastasis organs (162).

Exosomes are capable of carrying various ncRNAs to establish cancer development by promoting multiple aspects of tumor biology. Exosomal ncRNAs (also called ncRNA-exosomes) are known as key determinants of tumor fate and could be chased or manipulated to achieve clinical advantages (163). RNA products remain viable and conserve their potential in the exosomes while they travel outside the cell, where the major amount of miRNAs in circulation are packed in the exosomes (164). Noncoding RNA components of the exosomes are highly divergent according to the physiology of the tumor tissue and the cells from which they originate. All the ncRNAs’ subtypes along with other ncRNAs, are found in tumor-derived exosomes, influencing the condition of the recipient cells (165). Li and Tang (166) reported that exosomes containing miR-221-3p derived from M2 macrophages in ovarian cancer could enhance the cell cycle by facilitating G1/S transition as a result of CDKN1B downregulation. Also, by targeting PIK3R1, miR-221-3p augments resistance to adriamycin when delivered by exosomes to breast cancer cells (167). HOTAIR is a relatively well-known lncRNA that is dysregulated in diverse cancer types, including lung cancer. Exosomal HOTAIR derived from nonsmall cell lung cancer (NSCLC) cells enhanced cancer cell growth, migration, and invasion by acting as ceRNA for miR-203 (168). Recently, Xie et al. (169) concluded that delivery of exosomal circRNA vacuole membrane protein 1 (circVMP1) to cisplatin-sensitive NSCLC cells targets and sponges miR-524-5p and upregulates METTL3 and SOX2. The outcome was enhanced tumor resistance to cisplatin both *in vitro* and *in vivo* (169). It is also revealed that exosome-derived lnc01559 mediates gastric cancer (GC) stemness and metastasis, and its suppression leads to tumor inhibition. In addition, in a study by Xie et al. (169) in GC, exosomal circSHKBP1 inhibited miR-582-3p function to upregulate HUR and VEGF signaling the consequence of which was tumor progression (170). Another investigation designed by Yang et al. (171) identified the tumor-promoting role of exosomal hsa-circ-0085361 (circTRPS1) in bladder cancer. The study demonstrated that circTRPS1 delivered by exosomes is able to sponge and neutralize miR-141-3p, which in turn enhances glutamine metabolism via GLS1 overexpression. GLS1 upregulation could also induce CD8^+^ T-cell exhaustion and bladder cancer cell proliferation (171). Noncoding RNAs transferred in exosomes intensify tumor chemoresistance as well. For instance, exosomal lncRNA PART1 amplifies esophageal squamous cell carcinoma (ESCC) cells’ resistance to gefitinib. Regulation of miR-129/Bcl-2 pathway has been introduced as the underlying mechanism. Furthermore, PKM2 is reported to be upregulated in oxaliplatin-resistant colorectal cancer cells. Transportation of hsa-circ-0005963 via exosomes from resistant cells to sensitive counterparts and miR-122 neutralization were found to be the culprit machinery (148). The role of exosomal ncRNAs is also identified in the crosstalk between tumor cells and TME-residing macrophages and fibroblasts. Tumor-associated macrophages (TAMs) can switch phenotypes between M1 (proinflammatory) and M2 (anti-inflammatory) states in a reversible and dynamic manner. M2 macrophages secrete growth factors and inhibit antitumor immunity. Hepatocellular carcinoma (HCC) tissues are highly enriched with exosomal lncRNA TUC339, which induces M2 polarization in TAMs, decreased cytokine production and compromised phagocytosis. LncRNA TUC339 takes part in cytokine and chemokine receptor signaling pathways. Moreover, increased exosomal has-circ-0048117 secondary to hypoxia in ESCC tissue results in M2 polarization mediated by upregulating TLR-4 and miR-140 sponging. While in colon cancer, tumor-derived exosomal miR-21 causes M1 polarization by TLR-7 signal transduction, producing a suitable premetastatic niche in the liver (172, 173). Exosomes containing miR-501-3p, which originate from M2 TAMs, facilitate pancreatic cancer progression, invasion, and tube formation by targeting the *TGFBR3* gene and TGF-β pathway promotion (174). Intriguingly, cancer-associated fibroblasts (CAFs) are also modulated by exosomal ncRNAs. Metastatic HCC cells produce exosomes carrying miR-1247-3p in order to convert the normal tissue fibroblasts into CAFs by regulating B4GALT3 and β1-integrin-NF-κB activation in the pulmonary metastatic niche (175). All the mentioned example studies indicate that ncRNA-containing exosomes influence cancer behavior in multiple divergent ways, and profound apprehension of their aspects is crucial to acquiring a broader viewpoint toward the complexity of tumor setup.

## 7 ncRNA-exosomes and endometrial cancer: biology and applicable opportunities

ncRNAs, which are transferred by exosomes, regulate and foster the cancer hallmarks in gynecologic cancers, too. Investigations and multi-aspect study designs have been directed to elucidate the roles and tasks of ncRNA-exosomes in gynecologic solid cancers. However, the available data about these biological particles in EC pathogenesis is more limited (176). Similar to other cancers, EC tumor tissue cells communicate and affect the phenotypic features of each other by means of exosomes, which could be detected in the patients’ sera. Exosomes appear to be of great importance in the context of endometrial cancer. In a recent clinical plus *in silico* proteomic study by Sommella et al. (177), they suggested candidate exosomal proteins able to discriminate the patients from healthy controls with an acceptable sensitivity (178). Exosomal signal transduction participates in EC growth and proliferation, invasion, metastasis, and angiogenesis through diverse methods (179). Song et al. (180) proposed a model in which exosomes may promote EC growth and angiogenesis. They depicted that plasma exosomes from EC patients bear the capacity to induce tumor proliferation and human umbilical vein endothelial cell angiogenesis. The suggested mediator was exosomal lectin galactoside-binding soluble 3 binding protein (LGALS3BP), which caused EC growth and angiogenesis via the PI3K/AKT/VEGFA machinery pathway. Interestingly, patients with high tissue LGALS3BP had a limited survival period (180). The tumor physiology of EC is also regulated by the means of ncRNA-exosomes, and EC cells, TAMs, and CAFs exchange signals by these nanosized messenger vehicles (181). Zheng et al. (182) have reported that exosomal miR-93 is increased in amount in the EC patients’ bloodstream. More recently, Zhang et al. (183) revealed that this miRNA can target the *ZBTB7A* gene and promote EC. Therefore, exosomal miR-93 transduction could be considered an attempt by the tumor to stay viable and migrate to remote sites. It is worth indicating that miR-93 is sponged by lncRNA SNHG14, and SNHG14/miR-93/ZBTB7A is a functional tumor-repressor axis in the EC cells (182, 184). Like other tumor types, CAFs have positive effects on the development of EC. It is concluded by Li et al. (147) that in EC tissues, exosomes derived from CAFs bear fewer amounts of miR-148b than exosomes from normal fibroblasts. The authors also reported that miR-148b targets and downregulates the oncogene DNMT1. Thus, the loss of exosomal miR-148b is a tumor-promoting phenomenon in EC. By exogenously transfecting CAFs with miR-148b, this RNA molecule was delivered by CAF-derived exosomes to the EC cells and inhibited EMT and tumor invasion, bringing a potential approach in the field of EC treatment (185). miRNA-499a-5p depicts a lower expression level in EC tissues compared to the neighboring normal endometrial cells. By embedding it into the exosomes obtained from EC mesenchymal stem cells, Jing et al. (186) were able to repress EC proliferation and angiogenesis both in laboratory and animal settings. They also demonstrated that Vav guanine nucleotide exchange factor 3 (VAV3) gene neutralization by miR-499a-5p is the underlying mechanism (186). We previously stated that ncRNA-exosomes have the ability to alter the characteristics of TAMs in the TME. An *in vitro* investigation by Xiao et al. (187) showed that under hypoxic conditions, EC cells increase their exosome production, and the expression profile of exosome-associated miRNAs differs as well. There was a significant increase in the exosomal miR-21, and when transferred into the THP-1 monocyte cells, these cells depicted M2-like polarization, as confirmed by IL-10 and CD206 overexpression. The abundance of M2 macrophages in EC samples is correlated with tumor aggressiveness and poor prognosis (187). Moreover, Wang et al. (188) indicated that TAMs enhance EMT and inhibit apoptosis in EC cells, partly by means of exosome transduction. They revealed that miR-192-5p is expressed lowly in TAM-derived exosomes. The oncogene axis IRAK1/NF-κB was reported as the target for the miRNA. Therefore, overexpressed miR-192-5p in exosomes secreted by TAMs would be a promising tool to be absorbed by EC cells to exert anticancer activity (188). Aiming to determine the differentially expressed core genes, the related pathways, and their influence on the prognosis of EC patients, Shi et al. (189) designed an *in silico* study. Their results included a couple of genes from different pathways; among them was FOXL2, which depicted reduced expression in EC tissues associated with reduced patient survival. In addition to verification of the obtained data in EC cell lines, they also investigated exosomal ncRNAs secreted from the tumor cells. By knowing the fact that miR-133a targets FOXL2 gene transcript, they found increased levels of miR-133a in the exosomes derived from the EC cells, which could be absorbed by normal endometrial cells. These results suggest a potential regulatory process in the EC tumorigenesis mediated by miRNA-containing exosomes (189). It is conventionally believed that tumor infiltration with effector CD8^+^ T cells results in antitumor cytotoxicity and tumor degradation. Novel studies indicate that there are additional mechanisms for the tumor-repressing role of the CD8^+^ T cells like exosome transduction. The study by Zhou et al. (190) depicted that, as a consequence of estrogen signaling, miR-765 is downregulated in EC tissues compared to the normal endometrium. They also inferred that the PLP2 gene, which promotes EMT and EC cell migration via Notch signaling, is regulated by this miRNA. The outstanding finding of this research was the elevated levels of miR-765 in exosomes produced by CD45RO^−^CD8^+^ T cells. This evidence indicates the activity of CD8^+^ T cells in EC via the formation of exosomes enriched with miR-765 to restrict the oncogenic impact of estrogen (191). Recent evidence also supports the potential of exosomal miR-26a-5p released by EC tumor cells and uptaken by lymphatic endothelial cells to induce lymphatic vessel generation and promote lymphatic spread of EC via LEF1/c-Myc/VEGFA axis (192). One of the remarkable risk factors for EC tumorigenesis is the concomitant underlying polycystic ovarian syndrome (PCOS). In order to explore the probable role of exosomal ncRNAs in this association, Che et al. found that EC cells demonstrate high levels of migration and invasion when exposed to exosomes isolated from the sera of PCOS patients. MicroRNA-27a-5p was the most notably upregulated miRNA in those exosomes. Target-prediction databases, confirmed by further luciferase assay, brought SMAD4 as the target for miR-27a-5p. Thus, circulating exosome-derived miR-27a-5p facilitates EC progression via downregulation of SMAD signaling in PCOS patients (193). Recently, Pan et al. (194) intended to bring an approach based on ncRNA-exosomes to target EC cells. They purified and collected exosomes containing miR-503-3p secreted from human umbilical cord blood mesenchymal stem cells. They also revealed that miR-503-3p targets mesoderm-specific transcript (MEST) gene, which is a potential cancer promoter. After transfecting with exosomal miR-503-3p, the growth and biologic features of the EC cells were suppressed proposing a beneficial opportunity in the EC treatment (194).

Also, lncRNAs could be the cargo for exosomes to transduce signals in the TME. As discussed previously, CAFs bear notable tumor-promoting characteristics in the EC by releasing ncRNA-exosomes. LncRNA NEAT1 is upregulated in EC tissues, and its abundance is correlated with higher tumor proliferation capacity. Fan et al. (168) found that NEAT1 is overexpressed in CAFs derived from EC tissues compared to normal endometrial fibroblasts, and this discrepancy was also spotted in exosomes derived from those cells. By transferring exosomal NEAT1 to EC cells, an acceleration in tumor growth was observed. Moreover, the authors concluded that the tumor progression is due to the influence of NEAT1 on the miR-26a/b-5p/STAT3 axis by sponging the miRNA, which increases YKL-40 expression (195). Since a couple of years ago, lncRNA DLEU1 has been introduced as a mediator in EC cell viability and invasion. Recent research by Jia et al. (196) confirmed the upregulation and cancer-boosting role of this lncRNA in the EC tissues. They additionally stated that DLEU1 is transmitted by the exosomes to the adjacent EC cells and increases their capacity to proliferate and migrate. The underlying mechanism for this phenomenon is that DLEU1 targets and sponges miR-381-3p, which is an identified regulator of the E2F3 oncogene (196). LncRNA NONHAT076754 functions to facilitate metastasis and invasion in cancer. Qiu and Hua, (197) revealed that NONHAT076754 is highly detected in ectopic endometrial stroma relative to the normal eutopic endometrium in endometriosis patients. In addition, this lncRNA could be shuttled via exosomes from the ectopic cells and uptaken by the normal cells, resulting in an upregulation of invasion capacity in the recipients. The underlying mechanism was the regulation of the tight junction protein ZO-1, E-cadherin, and N-cadherin (197). Identification of exosomal circRNAs in cancers has shed more light on the mechanisms by which tumor biology is regulated. This concept, as well as other ncRNA-exosomes, may determine the clinical features or predict the EC patients’ prognosis. The profile of circulating exosomal circRNAs in EC patients and healthy controls is diverse. The study by Xu et al. (198) demonstrated 209 increased and 66 decreased exosomal circRNAs in the sera of EC patients. The major molecular pathways in which the dysregulated circRNAs took part were actin cytoskeleton regulation, ECM–receptor interaction, and focal adhesion pathways. However, increased fold changes for only two circRNAs, hsa-circ-0002577 and hsa-circ-0109046, were calculated to be greater than 2 (198). An interesting investigation by Gu et al. (199) depicted a model for the role of exosomal circRNAs as a communication tool for cellular members of the TME. According to their report, TAMs obtained from human EC tissues bear M2-like features, and TAM-derived exosomes absorbed by EC cells reduced radiosensitivity of the EC cells *in vitro*. Circular RNA microarray analysis indicated that hsa-circ-0001610 was the most abundant circRNA in the exosomes from TAMs. They also concluded that hsa-circ-0001610 was the mediator for the radioresistance of the EC cells via functioning as ceRNA for miR-139-5p. *In silico* and *in vitro* analyses suggested cyclin B1, a vital facilitator of radioresistance in multiple cancers, as the target for miR-139-5p. The mentioned data were confirmed in a xenograft tumor model study on nude mice. Thus, TAM-derived exosomal hsa-circ-0001610 contributes to radioresistance in EC by regulating the miR-139-5p/cyclin B1 axis (199). Eventually, as a novel approach in the field of ncRNA-exosome discovery in EC, Qian et al. (200) studied the expression profile of transfer RNA-derived small RNAs (tsRNAs) in tissue samples and serum specimens. They concluded that the expression of tRF-20-S998LO9D was significantly inhibited in both the tissue and serum exosomes of the patients. tRF-20-S998LO9D overexpression led to EC cell apoptosis as well as decreased proliferation, invasion, and migration capacity. However, the field of exosomal tsRNAs requires additional precise investigations (200).

Taken together, recent studies considering the prospects of ncRNA-exosomes in EC have brought encouraging results so far. Some studies have designated the ncRNAs transmitted by the exosomes as a part of the tumor physiology, while others have disclosed the possible applications of ncRNA delivery via exosomes with therapeutic intentions to alter the EC biology. **Table 1** summarizes the prominent details of the aforementioned studies about exosomal ncRNA transportation in EC.

**Table 1:** The transport of exosomal noncoding RNAs (ncRNAs) in endometrial cancer (EC).

ncRNA cargo	Origin	Target	Biological effect	Consequence	Ref.
miR-93	EC cells	EC cells	ZBTB7A gene regulation	Increased tumor proliferation and metastasis	Zheng et al. (182) and Zhang et al. (184)
miR-21	EC cells	TAMs	M2 polarization of TAMs	Increased tumor aggressiveness and poor patient prognosis	Xiao et al. (187)
miR-133a	EC cells	Normal endometrial cells	FOXL2 gene regulation	Increased tumorigenesis	Shi et al. (189)
miR-765	CD45RO^−^CD8^+^ T cells	EC cells	Regulation of PLP2/Notch axis	Decreased EMT and tumor cell migration	Zhou et al. (190)
miR-26a-5p	EC cells	Lymphatic endothelial cells	LEF1/c-Myc/VEGFA axis promotion	Lymphatic formation and tumor spread	Wang et al. (188)
miR-27a-5p	Bloodstream (*no identified cell*)	EC cells	SMAD4 gene regulation	Tumor progression in PCOS patients	Che et al. (193)
miR-148b*	CAFs	EC cells	DNMT1 gene regulation	Decreased EMT and tumor invasiveness	Liu et al. (201)
miR-499a-5p*	EC mesenchymal stem cells	EC cells	VAV3 gene regulation	Decreased tumor proliferation and angiogenesis	Jing et al. (186)
miR-192-5p*	TAMs	EC cells	Regulation of IRAK1/NF-κB axis	Decreased EMT and increased apoptosis	Wang et al. (188)
miR-503-3p*	Umbilical cord blood mesenchymal stem cells	EC cells	MEST gene regulation	Tumor suppression	Pan et al. (194)
lncRNA NEAT1	CAFs	EC cells	Regulation of miR-26a/b-5p/STAT3/YKL-40 axis	Increased tumor growth	Fan et al. (168)
lncRNA DLEU1	EC cells	EC cells	Regulation of miR-381-3p/E2F3 axis	Increased tumor proliferation and migration	Jia et al. (196)
lncRNA NONHAT076754	Ectopic endometrial cells	Normal endometrial cells	Regulation of ZO-1, E-cadherin, and N-cadherin	Increased invasion capacity	Qiu et al. (197)
hsa-circ-0001610	TAMs	EC cells	Regulation of miR-139-5p/cyclin B1 axis	Increased radioresistance	Gu et al. (199)

## 8 The role of exosomes in diagnosis, prognosis, and anticancer therapy

Exosomes belong to phospholipid bilayer extracellular vesicles, and they are released by all types of cells, including cancer cells. Exosomes contain DNA and RNA, lipids, and proteins and also perform communication functions between cells (202). As a result, exosomes have been used in the diagnosis and prognosis of endometrial cancer through the analysis of biomarkers, showed at **Table 2** constituting the content of exosomes. Moreover, the above diagnostics are specific and noninvasive (234). The first biomarker may be ncRNA, which includes miR-15a-5p (227). miR-15a-5p expression was consistently upregulated in plasma-derived exosomes from EC patients. Moreover, patients with a large tumor had higher exosomal miR-15a5p expression compared with a small tumor, which may indicate the significant usefulness of this biomarker in EC diagnostics (190). It is also worth mentioning that miR204-5p, miR-423-3p, and miR-20b-5p, miR-143-3p, and miR-195-5p expression were increased in the serum of EC patients. miR204-5p could suppress cancer by suppressing EMT, and self-renewing cancer stem cells. miR-423-3p may participate in the development and progression of EC, namely inhibiting cisplatin-induced apoptosis. miR-20b-5p could modulate vascular endothelial growth factor A (VEGFA) transcription by targeting hypoxia-inducible 4 factor 1-alpha (HIF1α), may act as a tumor suppressor by hindering MMP-2 expression, leading to cell cycle arrest, and also has a regulatory function in oxygen balance (195). Another biomarker, miR-143-3p, can inhibit cell proliferation and EC metastasis through mitogen-activated protein kinase (MAPK1) (235). miR-195-5p-bound signaling pathway target genes involved in oncogenic mechanisms, such as cell proliferation and apoptosis (234). miR-205 has increased expression in the EC compared to normal endometrial tissues; mutations in miR-205 were found in EC for 25%–83% of cases. miR-205 participates in regulating the expression of PTEN, which is the most common mutated tumor suppressor gene. PTEN takes part in inhibitory function by proliferation and promoting apoptosis, deletion, or mutation leading to carcinogenesis. miR-205 upregulation blocked PTEN translation and activated the AKT pathway; constitutive activation of AKT contributes to tumor progression. It should also be noted that miR-205 acts as an oncogene and inhibits cellular apoptosis in the EC by targeting the PTEN/AKT pathway. Also, miR-205 plays a significant role in the invasion and immigration of endometrial cancer. This mechanism is based on the targeting of miR-205 to the AKT pathway by inhibition of E-cadherin expression, promotion of Snail expression, and downregulation of glycogen synthesizing kinase 3β (236). Circular RNAs (circRNAs) also belong to noncoding RNA and may be a useful diagnostic and prognostic tool; specifically, they are hsa_circ_0109046 and hsa_circ_0002577. Increased expression of circRNAs was demonstrated in EC. circRNAs are related to, e.g., neoplastic migration, the ECM–receptor interaction pathway, the regulation of actin cytoskeleton pathway, and the focal adhesion pathway (198). It is also worth emphasizing that increased expression of proteins such as metalloproteinase 9 (MMP9) and pyruvate kinase (KPYM) has been found in EC patients and showed 94% sensitivity and 87% specificity for EC (237). The NEAT1 (a long noncoding RNA) participates in invasion, chemoresistance in EC cells, and remodeling tumor microenvironment by induced miR-361 suppression, which activates STAT3. Higher levels of NEAT1 may be positively correlated with advanced tumor stage and lymph node metastasis. Another important biomarker is WNT7A, coding protein Wnt-7a. The expression of WNT7A was higher in EC tissue than in normal endometrial tissue. Furthermore, increased WNT7A expression was associated with a high tumor grade, increased depth of myometrial invasion, lymph node metastasis, and vascular invasion (100). LGALS3BP may be a biomarker with diagnostic and prognostic value that causes EC growth and angiogenesis by the PI3K/AKT/VEGFA machinery pathway (180).

**Table 2:** List of commonly observed markers in research on endometrial cancer.

EC marker	Also known as	Identification: RefSeq; Gene ID	Family	Linked cancers	Research in EC
Human epididymis protein 4 (HE-4)	WAP 4-disulfide core domain protein 2 (WFDC2)	NC_000020.11; 10406	WFDC	Ovarian, endometrial, lung	Chen et al. (203), Das et al. (204), Barr et al. (205), and Yue et al. (206)
Cancer antigen 125 (CA-125	Mucin-16 (MUC-16); ovarian cancer-related tumor marker CA125	NC_000019.10; 94025	*Mucins*	Ovarian, breast, lung, colon, gallbladder	Chen et al. (203), Zhai et al. (207), Cheng et al. (208)
Cancer antigen 15-3 (CA15-3)	Mucin-1 (MUC-1)	NC_000001.11; 4582	*Mucins*	Duodenal, breast, colorectal, lung	Sezgin et al. (209), Gök et al. (210)
CD40 molecule (CD40)	Bp50, CDW40, TNFRSF5, p50	NC_000020.11; 958	TNF receptors	Liver, rectal, pancreatic ductal, cutaneous, lung	Thavaneswaran et al. (211), Huang et al. (212), and Zhao et al. (213)
Phosphatase and tensin homolog (PTEN)	10q23del, mutated in multiple advanced cancers (MMAC)	NC_000010.11; 5728	Protein-tyrosine phosphatase (PTP)	Gastric, breast, prostate, pancreatic, colorectal, ovarian	Moroney et al. (214), Raffone et al. (215), and Hu et al. (216)
Carcinoembryonic antigen cell adhesion molecule 1 (CAECAM1)	Cluster of differentiation 66a (CD66a)	NC_000019.10; 958	CEA	Thyroid, esophageal, bile duct, breast, lung, pancreatic	Bamberger et al. (217), Li et al. (218), and Ge et al. (219)
Chitinase-3-like protein 1 (CHI3L1)	Cartilage glycoprotein 39 (CGP-39); YKL-40	NC_000001.11; 1116	GH18	Colorectal, ovarian, pleural, brain, pancreatic, esophageal	Song et al. (220), Fan et al. (221), and Unuvar et al. (222)
Serum amyloid A1 (SAA1)	TP53I4; PIG4	NC_000011.1; 86288	SAA	Ovarian, liver, glial, pancreatic, endometrial	Omer et al. (223) and Ren et al. (224)
Lipocalin-2 (LCN-2)	Oncogene 24p3; neutrophil gelatinase-associated lipocalin (NGAL)	NC_000009.12; 3934	Lipocalins	Cervix, esophageal, pancreas, kidney, liver	Jiang et al. (225) and Su and Yin (226)
Vascular endothelial growth factor (VEGF)	Vascular permeability factor (VPF)	NC_000006.12; 7422	PDGF	Prostate, gaster, mammary gland, ovarian, kidney, breast colorectal	Dong et al. (227), Jimeno et al. (228), and Guan and Zhang (229)
Interleukin-11 (IL-11)	Adipogenesis inhibitory factor (AGIF)	NC_000019.10; 3589	Interleukins	Colorectal, lung, liver, oropharyngeal	Wang et al. (51) and Hassani et al. (230)
Programmed death receptor-1 (PD-1)	Cluster of differentiation 279 (CD279)	NC_000002.12; 5133	CD28/CTLA-4	Lung, colorectal, kidney, bone, gastric	Lam et al. (231), Ducceschi et al. (232), and Goodman et al. (233)

Exosomes have been used not only in diagnosis and prognosis but also as therapeutic tools in the treatment of EC due to their properties that play a key role in the communication between the tumor cells and the tumor microenvironment EC, and they can also be collected from various body fluids such as uterine fluid and blood. As a result, they provide a noninvasive method for diagnosing and monitoring endometrial cancer. Exosomes contain a cargo that may constitute tumor suppression by adding tumor-suppressive miRNAs or proteins to inhibit their growth and metastasis cells of EC (238). Engineered exosomes with cargo tumor-suppressor miRNAs may become a useful therapeutic tool in the fight against EC cancer progression. miR-499a-5p inhibits EC proliferation by targeting VAV3 and suppresses EC growth, metastasis, angiogenesis, or metastasis (186). It should also be noted that human umbilical cord mesenchymal stem cell (hUCMSC)-derived exosomes may be a valuable tool for target-based therapies, namely engineered exosomes that, with tumor-suppressor miRNAs, can be delivered by hUCMSCs to EC cells and finally inhibit their proliferation (239) targeted EC cells overexpressing miR-302a exosomes and disrupted their migration proliferation and by suppressing cyclin D1 levels and inactivating the AKT signaling pathway (240). Additionally, overexpression of miR-503-3p in hUCMSC-derived exosomes inhibited tumor growth and suppressed the expression of MEST, which is involved in EC by cell proliferation, differentiation, and apoptosis (181) Another application example is exogenously transfected miR148b cancer-associated fibroblasts (CAFs)-derived exosomes, which suppress EC cell invasion and metastasis by targeting *DNMT1* gene. DNA (cytosine-5)-methyltransferase 1 (Dnmt1) in humans is encoded by the *DNMT1* gene. Dnmt1 takes part in DNA methylation. Aberrant methylation patterns are associated with certain human tumors, including EC (185). The direct transfer of CAF-secreted exosomal miR-320a to EC cells inhibited their invasion, proliferation, and migration by targeting the HIF1α–VEGFA axis. MiR-320a binds directly and regulates the mRNA of HIF1α. HIF1α is known to target the vascular endothelial VEGFA (184). MSC-derived exosomes loaded with miR-499a-5p could suppress tumor growth migration, invasion, or angiogenesis of EC cells by targeting VAV3, which is a guanine nucleotide exchange factor (GEF) that regulates the activity of Rho/Rac family GTPases. The main role is the regulation of cell differentiation, motility, and proliferation (186).

## 9 Future perspectives

There is still not enough research on specific markers for EC, despite the growing interest in treating endometrial cancer. Some markers, such as CA-125 or HEP-4, are not specific only to EC. They can give false-positive results, especially in the presence of pelvic inflammation or other gynecological cancers or endometriosis. Another limitation is the absence of elevated marker levels in the early stages of the disease. This makes early diagnosis impossible. Unfortunately, marker levels can also change in response to treatment, which also makes it difficult to interpret the results.

EC shows quite significant genetic variability, which also contributes to problems in finding universal markers. Another challenge is the variability of this type of cancer and the problem of diagnosis. Due to the use of mostly invasive methods of EC treatment, it is worthwhile to look into noninvasive technologies. Magnetic resonance imaging with molecular contrasts can aid in the early detection and assessment of tumor extent. Despite the limitations of markers, research in this direction continues. The investigation of genetic and epigenetic markers shows great specificity and sensitivity. Metabolic markers also seem very promising; this is related to their frequent variability in the very early stages of the disease. This would favorably affect the problems of late diagnosis of this type of cancer, increasing the chances for patients.

CAR-T research, which is developing quite rapidly in the context of endometrial cancer, also seems promising. A key factor in this therapy is the identification of specific antigens for endometrial cells that are not present in healthy cells. Increasing the safety of the therapy by using molecular “breakers” also seems promising. This minimizes the risk of cytokine release syndrome as well as other toxicities. Another limitation may be the costs associated with conducting such studies, as well as their length and complexity. It is also worth mentioning tumor-infiltrating lymphocytes, these are lymphocytes isolated directly from the tumor, multiplied and activated *in vitro*, and then reintroduced into the patient’s body. This is a type of adoptive cell therapy that supports the patient’s immune system in the fight against cancer. This therapy is promising due to its low toxicity. However, there are still limitations, mainly related to isolation time, cell multiplication, and cost. Unfortunately, the effectiveness of this therapy is mainly dependent on the number of isolated lymphocytes.

Researchers suggest a potential role for p53 as an antigen in the acquired immune response and as a key monitor of the innate immune system. The use of antibodies against p53 is expected to be therapeutically effective against cancers carrying the same p53 mutant. In recent years, more and more promising approaches to p53-based therapy have emerged, which may suggest its efficacy. The molecular classification of EC, consisting of ultra-mutated POLE, mismatch repair deficiency, p53 deficiency, and subgroups with nonspecific molecular profile (NSMP), has strong and independent prognostic value, including potential response to chemotherapy.

Exosomes and miRNAs also show great and crucial potential. The advantage of using exosomes may be primarily the lack of invasive monitoring methods, as they can be extracted from body fluids. They can also carry small RNAs that target genes responsible for cancer progression. They may also find use in carrying anticancer drugs directly to EC cells without adversely affecting healthy cells. However, there are also limitations to using this therapy. It is necessary to invent new isolation techniques that are more precise. In recent years, interest in the use of exosomes and miRNAs in endometrial cancer has been growing and may prove to be a breakthrough in the treatment of this problematic type of cancer.

The fact that there is no up-to-date classification of endometrial cancer worldwide is also a very serious limitation. There is very little information newer than 2022. It would be tremendously helpful to create a newer classification, which would certainly update the data.
